# Supplementary material for: Identification of mouse CD4+ T cell epitopes in SARS-CoV-2 BA.1 spike and nucleocapsid for use in peptide:MHCII tetramers
Source: Front Immunol. 2024 Mar 11;15:1329846. doi: 10.3389/fimmu.2024.1329846 (PMC10961420; doi:10.3389/fimmu.2024.1329846)
Supplement: Supplementary file 1 [file DataSheet_1.docx]

**Figure S1. Identification of lost epitopes in HexaPro spike**

**A)** Peptides were generated covering the regions of spike that were affected by proline substitutions in the HexaPro construct. Native residues that were replaced by prolines are indicated in red. **B)** C57BL/6 mice were immunized s.c. with a mix of the 4 peptides plus CFA as adjuvant and 9-10 days later, CD4^+^ T cells were tested for reactivity to each individual peptide by IFNγ ELISpot assay. Mean values ± SEM are shown for n=2-6 mice processed across multiple independent experiments. **C)** Representative flow cytometry plots of CD4^+^ gated events illustrating S-883 tetramer staining of epitope-specific T cells from naïve and S-883 peptide-immunized mice. **D)** Quantification of S-883-specific CD4^+^ T cells from naïve and peptide-immunized mice. Mean values ± SEM are shown for n=5-6 mice per epitope across multiple independent experiments. The dotted line represents the limit of detection as deﬁned by the mean numbers of CD8^+^tetramer^+^ events per mouse. Statistical significance was calculated via Mann-Whitney test; p = 0.13.

**Figure S2. Analysis of CD4^+^ T cell epitopes in the ancestral strain of SARS-CoV-2, but not BA.1**

Peptides representing Wuhan spike (70 peptides) and nucleocapsid (9 peptides) sequences covering regions of the protein that were mutated in BA.1 were generated. C57BL/6 mice were immunized s.c. with Wuhan spike **(A)** or nucleocapsid **(B)** proteins plus CFA as adjuvant and 9-10 days later, CD4^+^ T cells were tested for reactivity to each individual peptide by IFNγ ELISpot assay. Mean values ± SEM are shown for n=3 independent experiments. **C)** Representative flow cytometry plots of CD4^+^ gated events illustrating S-63 tetramer staining of epitope-specific T cells from naïve, S-63 peptide-immunized, or Wuhan spike protein immunized mice. **D)** Quantification of S-63-specific CD4^+^ T cells from naïve, peptide immunized, and protein immunized mice. Mean values ± SEM are shown for n=5-9 mice per epitope across multiple independent experiments. The dotted line represents the limit of detection as deﬁned by the mean numbers of CD8^+^tetramer^+^ events per mouse. Statistical significance was calculated via Kruskal-Wallis tests with Dunn`s multiple comparison of each immunized set to naive; **p<0.01.
